# Seroprevalence of cystic echinococcosis in a high-risk area (Al-Mafraq Governorate) in Jordan, using indirect hemagglutination test

**DOI:** 10.1016/j.parepi.2019.e00104

**Published:** 2019-04-11

**Authors:** Nisreen Himsawi, Nawal Hijjawi, Ali Al-Radaideh, Mohammad Al-Tamimi

**Affiliations:** a Department of Medical Laboratory Sciences, Faculty of Allied Health Sciences, Hashemite University, Zarqa, Jordan; b Department of Medical Imaging, Faculty of Allied Health Sciences, Hashemite University, Zarqa, Jordan; c Department of Basic Medical Sciences, Faculty of Medicine, Hashemite University, Zarqa, Jordan

**Keywords:** Cystic Echinococcosis (CE), Hydatid disease (HD), *Echinococcus granulosus*, Jordan, Indirect hemagglutination (IHA), Seroprevalence

## Abstract

Hydatid disease (HD) is a zoonotic disease of humans and animals which is caused by infection with the larval stages of the taeniid cestodes of the genus *Echinococcus*. HD is endemic in many countries of the Middle East, including Jordan. The seroprevalence rate of HD in areas of elevated risk in Jordan has not previously been investigated using indirect haemagglutination (IHA) testing. In the present study, 512 blood samples were collected from recruited outpatients from an internal medicine clinic in Al-Mafraq Governmental Hospital in Jordan. Each participant signed a consent form and completed a questionnaire. The presence of antibodies specific for *E. granulosus* antigens was detected using an IHA test. Statistical analysis was performed by SPSS software using the Chi-square test. In all, 4.1% of the study participants were seropositive for *E. granulosus* IgG antibodies. There was a significant correlation between unexplained weight loss among seropositive patients (*P* = 0.018). Seropositivity was significantly higher in patients who slaughtered sheep inside their houses (*P* = 0.023). HD seroprevalence did not correlate with gender (*P* = 0.433), age (*P* = 0.880), residency status (*P* = 0.938), or educational level (*P* = 0.808). The vast majority (75.2%) of participants reported no prior knowledge about HD, and 99.8% were not aware about the etiology of the disease.

## 1 Introduction

Hydatid disease (HD), or cystic echinococcosis (CE), is a worldwide zoonotic disease which is caused by infection with the larval stage of the cestode tapeworms of the species *Echinococcus granulosus* (Moro and Schantz, 2009). Hydatid disease affects over 1 million people globally (Higuita et al., 2016). CE is transmitted between the definitive host (dogs or other canids) and the intermediate hosts (herbivorous and omnivorous). Humans and other intermediate hosts acquire HD upon swallowing the eggs of the *E. granulosus* parasite in contaminated food or water (Moro and Schantz, 2009). Hydatid disease is usually asymptomatic, but it can present clinically as complicated unilocular cysts. The most frequent complication of HD is the periparasitic organ compression upon cyst growth or cyst rupture (Craig et al., 2003). Multiple risk factors are known to play important roles in HD prevalence and transmission, including dog ownership, illiteracy, farming and shepherd in, and residency in rural areas or with nomadic populations (Moro and Schantz, 2009; Higuita et al., 2016; Craig et al., 2003).

High prevalence of HD in human and animal hosts is usually observed in countries of the temperate zones, including several parts of Eurasia (Mediterranean regions, southern and central parts of Russia, central Asia, China, Australia, some parts of America (South America), and north and east Africa) (Grosso et al., 2012). In the Middle East, the prevalence of HD varies from country to country. For example, the annual prevalence recorded by Al-Maqased Hospital in Jerusalem was 1.76 per 100,000 inhabitants (Abdel-Hafez and Kamhawi, 1997), while in hospitals of the Palestinian West Bank, the prevalence was 3.1 per 100,000 inhabitants (Abu-Hasan et al., 2002). In an Egyptian retrospective study, the annual incidence of HD ranged between 1.34 and 2.60 per 100,000 inhabitants (Kandeel et al., 2004). A recent metaanalysis of HD seroprevalence in the Middle East, using 53 studies over 3 decades with a total population of 17,775 individuals, indicated an overall seroprevalence of 7.44%, with the highest seroprevalence reported for Turkey (34.6%) and the lowest seroprevalence reported for Oman (0.3%) (Galeh et al., 2018). HD is endemic in many regions of Jordan as reported by several studies and case reports since 1966 (Hijjawi et al., 2018; Al-Qaoud et al., 2003a). The seroprevalence of HD among Jordanians was investigated by two comprehensive studies. The first study indicated a seroprevalence rate of 2.4% (Qaqish et al., 2003), and the second study indicated higher and more variable seroprevalence rates that ranged from 11.4% among the rural-agricultural subjects to 5.0% in the semi-Bedouin population to 3.7% in the Bedouin population (Qaqish et al., 2003). The general Jordanian population has an overall seroprevalence rate of 3.5% (Galeh et al., 2018).

Hydatid disease is usually diagnosed incidentally during routine radiological imaging (Srinivas et al., 2016). Ultrasound (US) is considered the gold standard method for HD diagnosis due to its availability and low cost (Caremani et al., 1997). Computed tomography (CT) scanning has a higher sensitivity and specificity than US in the diagnosis of HD (Stojkovic et al., 2012). Magnetic resonance imaging (MRI) can also demonstrate cyst wall defects (Stojkovic et al., 2012; Brunetti et al., 2010). In Jordan, most retrospective studies and case series have shown that US and CT scanning have commonly been used in the diagnosis of HD (Al-Qaoud et al., 2003a; Al-Radaideh et al., 2017). Different serological assays are available to confirm the HD diagnosis including ELISA and IHA tests. Immunodiagnosis is useful for primary diagnosis of HD and for the follow-up of patients after surgical and pharmacological treatments (Zhang et al., 2011; Zhang et al., 2003). IHA assay is easier to perform, better established, less expensive, and faster than ELISA with a higher sensitivity and specificity (Van Gool et al., 2002; Al-Qaoud et al., 2003b; Ajlouni et al., 1984; Abo-Shehada, 1993; Governorate A-M., 2018).

Previous reports of the prevalence of HD in Jordan were based on surgical records and single case studies, and they therefore overlooked the asymptomatic cases and this could underestimate the actual prevalence/infection rate of the disease in the country. Two old seroprevalence studies in Jordan were conducted using ELISA (Qaqish et al., 2003). Al-Mafraq governorate is considered as a high risk area for HD due to its low socioeconomic and educational status, the abundance of agricultural and sheep raising workers, its high rates of stray dogs and sheep (Kayaalp et al., 2011; Kamhawi, 1995; Moosa and Abdel-Hafez, 1994; Lamb, 2012). The seroprevalence rate of HD in Al-Mafraq city has not been investigated before. Therefore, the aim of the present study was to estimate the seroprevalence rate of HD by testing patients visiting the internal medicine clinic of Al-Mafraq Governmental Hospital using IHA test.

## 2 Materials and methods

### 2.1 Study participants

A total of 512 patients referred to the internal medicine clinic of Al-Mafraq Governmental Hospital, Al-Mafraq city, Jordan, from December 2016 to May 2017, were recruited. Each participant signed a consent form to participate in the study and completed a questionnaire with his/her demographic details and health background. Participants were adults (age ≥ 18) from both genders. The study protocol was approved by the Institutional Review Board at the Hashemite University and the Jordanian Ministry of Health.

### 2.2 Serum samples

Under aseptic conditions, venous blood samples were collected individually from each participant in plain tubes. Blood was allowed to clot at room temperature for 15–30 min before being centrifuged (at 3000*g*, 20 °C for 15 min) to separate serum. Serum samples were stored at −20 °C for further immunological analysis. Serum samples from six histologically confirmed HD patients (with different antibody titers) were obtained from the Jordan University Hospital to serve as positive controls. Serum samples from healthy donors were used as negative controls.

### 2.3 Indirect hemagglutination test (IHA)

The *Echinococcus* IHA commercial kit (ELITech, kit number 66604, France) was used according to the manufacturer instructions (ELITECH, 2017) with a slight modification which involved the replacement of the U-shaped microtiter plates with V-shaped microtiter plates (Greiner Laboratories, Alphen, The Netherlands) in order to improve the visualization of the results (Van Gool et al., 2002). Briefly, fifty microliters of a 1:40 initial dilution of each serum sample was subjected to twofold serial dilutions to reach the final dilution of 1:1256. One drop of sheep RBCs sensitized with *E. granulosus* antigen (adult worm antigen) was added to each diluted sample. Positive and negative control sera were included in each test. After incubation for 2 h at room temperature, the titer of the tested serum was recorded as one dilution, which yielded a clear, sharp dark spot similar to those in the negative control wells. Titers were expressed as reciprocal values and the results were evaluated with a cutoff titer of 1:160 as recommended by the manufacturer. The antigen used in IHA is antigen 5, and it is primarily recognized by IgG1 in the patient's serum.

### 2.4 Statistical analysis

Data analysis was performed using SPSS version 20 software. A chi-square test was used to examine the relationship of demographic data, symptoms, and risk factors with immunodiagnostic test results. The test was considered significant if the *P*-value was <0.05.

## 3 Results

### 3.1 Characteristics of study participants

All recruited participants (*n* = 512) were residents of Al-Mafraq city and its surrounding areas and were referred to the internal medicine department of Al-Mafraq Governmental Hospital. All participants were Jordanian, except eight who were Syrian refugees (1.6%). There were 67.4% (*n* = 345) of the study participants who were female with a mean age of 41.7 years and 32.6% (*n* = 167) who were males with a mean age of 49.1 years. Most participants, 84% (*n* = 430), were married, and 45.5% (*n* = 233) did not complete their secondary education (Table 1).

**Table 1:** Demographic distribution of the study participants (*n* = 512).

Variable	Category	Number	Frequency %
Gender	Male	167	32.6
	Female	345	67.4
Marital status	Single	68	13.3
	Married	430	84.0
	Widow	11	2.1
	Divorced	3	0.6
Age (year)	18–30	91	17.8
	31–51	269	52.5
	52–80	152	29.7
Nationality	Jordanian	504	98.4
	Syrian	8	1.6
Address	Al-khalidia	75	14.6
	Al-hay alsharqi	53	10.4
	Al-hay aljanubi	58	11.3
	Hay al-hussein	49	9.6
	Other surroundings areas	277	54.1
Education level	PhD	3	0.6
	High diploma	5	1.0
	Master	4	0.8
	Bachelor's	82	16.0
	Diploma	50	9.8
	Secondary school	135	26.4
	<Secondary school	233	45.5

### 3.2 Seroprevalence of HD by IHA

Among the recruited participants, 4.1% (21/512) tested positive for *E. granulosus* IgG antibodies using IHA testing. The prevalence of HD was higher in males compared to females without being a statistically significant difference (χ^2^ = 0.615, *P* = 0.433). Slightly higher prevalence was observed in participants aged 31 to 51 years without achieving statistical significance (4.46%, χ^2^ = 0.256, *P* = 0.880). There was no significant difference in the seroprevalence of HD in the rural (surrounding) areas around Al-Mafraq city compared to the city itself (χ^2^ = 0.803, *P* = 0.938), and there was no correlation between educational level and positive IHA (χ^2^ = 3.008, *P* = 0.808) (Table 2).

**Table 2:** HD IHA seroprevalence according to participant gender, age category, region of residency and educational level.

Variable	Category	Positive IHA Number and (%)	χ^2^	*P* value
Gender	Male Female	9/167 (5.39) 12/345 (3.48)	0.615	0.433
Age (year)	18–30 31–51 52–80	3/91 (3.29) 12/269 (4.46) 6/152 (3.94)	0.256	0.880
Region	Al-khalidia Al-hay alsharqi Al-hay aljanubi Hay al-hussein Other surroundings areas	4/75 (5.33) 3/53 (5.66) 2/58 (3.44) 2/49 (4.08) 10/277 (3.61)	0.803	0.938
Education level	PhD High diploma Master Bachelor's Diploma Secondary school <Secondary school	0/3 (0.0) 0/5 (0.0) 0/4 (0.0) 3/82 (3.65) 1/50 (2.0) 6/135 (4.44) 12/233 (5.15)	3.008	0.808

### 3.3 Seropositive IHA correlation with HD symptoms and risk factors

Several symptoms relevant to clinical manifestations of hydatid disease were included in the questionnaire. No symptoms possessed a significant relationship with IHA seropositivity except unexplained weight loss, which showed significant correlation (χ^2^ = 5.576, *P* = 0.018) (Table 3). The correlation between possible risk factors for hydatid disease and positive IHA indicated a positive correlation between slaughtering sheep inside the house and positive IHA (χ^2^ = 5.197, *P* = 0.023). Correlation between all other possible risk factors, including previous cyst removal, eating raw vegetables, working on farms, having pets such as dogs or sheep at home, slaughtering sheep outside the home, and feeding the dogs with slaughtered animal remnants, with positive IHA were not significant (Table 4).

**Table 3:** Association between positive IHA and clinical symptoms.

Symptom	χ^2^	*P* value
Headache	0.219	0.640
Weakness and fatigue	0.467	0.494
Shortness of breath	0.178	0.673
Abdominal distension	0.000	1.00
Diarrhea	0.000	1.00
Vomiting	0.030	0.862
Unexplained weight loss	5.576	**0.018**
Stomach disorders	0.053	0.818
Cough	0.000	1.00
Anemia	0.000	1.00
Jaundice	1.367	0.242

**Table 4:** Association between possible risk factors and positive IHA.

Risk factor	Answer	Positive IHA Number and (%)	χ^2^	*P* value
History of cyst removal	Yes No	2 (0.4) 510 (99.6)	2.229	0.135
Eating raw vegetables	Yes No	21 (4.1) 491 (95.9)	0.000	1.000
Working at a farm or having pets at home	Yes No	4 (0.8) 508 (99.2)	0.000	1.000
Rising dogs inside house	Yes No	52 (10.2) 460 (89.8)	1.017	0.313
Rising sheep inside house	Yes No	64 (12.5) 448 (87.5)	0.348	0.555
Slaughtering sheep inside house	Yes No	164 (32) 348 (68)	5.197	**0.023**
Slaughtering sheep outside house	Yes No	117 (22.9) 395 (77.1)	0.138	0.710
Feeding dogs slaughtered animals remnants	Yes No	108 (21.1) 404 (78.9)	2.812	0.094

### 3.4 Knowledge and attitude toward HD

In all, 75.2% (385/512) of total participants indicated no prior knowledge about hydatid disease. Additionally, 96.1% (492/512) denied knowledge of people infected with hydatid disease, and 99.8% (511/512) reported no prior knowledge about the etiology of hydatid disease.

## 4 Discussion

Hydatid disease is a serious zoonotic infection which is prevalent in many countries worldwide including Jordan (Al-Qaoud et al., 2003b; Ajlouni et al., 1984). Exposure to the *Echinococcus granulosus* parasite eggs secreted in the feces of infected stray dogs plays an essential role in disease transmission (Ajlouni et al., 1984; Abo-Shehada, 1993). Determination of prevalence rate, common symptoms, and risk factors is important to estimate the significance and route of transmission of HD among certain populations. As HD usually starts with a relatively long asymptomatic infection phase, a reliable screening method would be valuable for early detection of the infection in endemic areas, thus allowing to treat infected individuals prior to severe disease manifestation (Craig et al., 2003).

Al-Mafraq Governorate, located in the northeastern part of Jordan, is the second largest governorate in the country which is 80 km far away from the capital Amman. It has 549,948 inhabitants over an area of 26,551 km^2^. Al-Mafraq Governmental Hospital is the referral center and the only tertiary facility in Al-Mafraq city (Abo-Shehada, 1993; Governorate A-M., 2018). Al-Mafraq Governorate population is a mixture of urban and rural populations with low socioeconomic and educational levels. Most of its residents work in agriculture and sheep raising (Jacob et al., 2016). Multiple studies have indicated that a higher prevalence rate of *E. granulosus* was observed in dogs and herbivores in northern Jordan (Hijjawi et al., 2018; Kayaalp et al., 2011; Moosa and Abdel-Hafez, 1994). Al-Mafraq city has hosted the largest number of Syrian refugees due to its proximity to Syrian-Jordanian border, which added significant strain to available health facilities (Moosa and Abdel-Hafez, 1994; Sarkari and Rezaei, 2015).

Large number of stray dogs is usually observed in the rural areas of Al-Mafraq Governorate, especially after the Syrian crisis where dogs escape and pass the borders and reside in these areas. Moreover, poor sanitation and hygiene in addition to low education status taken together, these factors could possibly increase the prevalence of HD in this specific area consistent with previous observations in other studies (Qaqish et al., 2003; Kayaalp et al., 2011; Kamhawi, 1995). The aim of this study focused on the assessment of the epidemiological seroprevalence of hydatid disease in high risk area “Al-Mafraq Governorate”. The research recruited a relatively large number of participants in order to enhance the ability of the sample in representing this specific community.

In the present study, the seroprevalence of HD as determined by IHA testing among the study participants was 4.1%. This rate is higher than the seroprevalence rates observed in two previous studies which reported an overall seroprevalence of 2.8% among school children and University students in northern and central Jordan (Abo-Shehada, 1993) and 2.4% among the general Jordanian population (Moosa and Abdel-Hafez, 1994). Furthermore, this rate is higher than the overall seroprevalence rate of 3.5% in the general Jordanian population calculated in a metaanalysis (Galeh et al., 2018). Al-Mafraq city has the highest seroprevalence rate among cities in Jordan, where the lowest rate was observed in Azraq city (2.5%) and the highest rate was observed in Amman city (3.5%) (Al-Qaoud et al., 2003a; Qaqish et al., 2003). The seroprevalence rate observed in the present study is lower than the rate reported by Qaqish and colleagues, who reported an overall seroprevalence rate of 7.7%, ranging from 11.4% in the rural–agricultural groups to 5% in the semi-Bedouin groups to 3.7% in the Bedouin groups (Qaqish et al., 2003). Differences in seroprevalence rate among different studies could be attributed to differences in sample size, geographic location, population under study, associated risk factors, and immunological assay used (Al-Qaoud et al., 2003a; Qaqish et al., 2003). All participants of this study were recruited from Al-Mafraq Governorate, thus the seroprevalence rate obtained is representative of this specific population within Jordan. Overall, Al-Mafraq Governorate has a high seropositive rate of HD and represents an area with higher risk for people to acquire the disease.

The advantages of using an IHA assay include its high sensitivity and specificity, simplicity, low cost, and short time frame for the results to be observed (Van Gool et al., 2002; Al-Qaoud et al., 2003b; Ajlouni et al., 1984; Abo-Shehada, 1993). The sensitivity of IHA is higher in the early stages of the disease and can range from 60 to 100%, with an average of 83%. A few studies have indicated that using ELISA for HD serodiagnosis and follow up might be more sensitive compared to IHA (Lamb, 2012). The specificity of IHA with reference to other non-cestode infections is usually high (95–100%) in spite of significant levels of cross-reactivity that might exist within the main larval parasitic infections, like fascioliasis and schistosomiasis (Sarkari and Rezaei, 2015). The IHA assay has a well-established role in the diagnosis of viral and other infectious diseases (Jacob et al., 2016). This is the first study to determine the seroprevalence rate of HD in Jordan using IHA testing (Qaqish et al., 2003; Srinivas et al., 2016). Western blot (WB) testing is regarded as a highly specific method but with low sensitivity for the diagnosis of HD (Liance et al., 2000). Differences in the seropositivity rate between IHA and WB might correlate with HD stage, viability and cysts fertility (Liance et al., 2000; Aslan et al., 2011).

The seroprevalence of HD in this study did not differ significantly in males compared to females, in agreement with another study in Jordan which reported no significant difference in HD seroprevalence between genders (Qaqish et al., 2003). Some studies showed a significant increase of HD prevalence in males (Qaqish et al., 2003; Gulsun et al., 2010). While other studies indicated a significantly increased prevalence rate in females (Abu-Hasan et al., 2002; Al-Radaideh et al., 2017; Abo-Shehada, 1993; Maktoof and Tabeekh, 2015; Nasrieh et al., 2003; Nasrieh and Abdel-Hafez, 2004; Zahawi et al., 1999), A meta-analysis showed no statistically significant differences between male and female individuals (male 9.3% and female 9.2%) in HD seroprevalence in the Middle East (Galeh et al., 2018). Differences in HD prevalence rate among genders could be related to gender specific differences in life style and occupation. HD seroprevalence did not differ significantly among the different age categories in this study, in consistency with other studies, where HD remains asymptomatic for many years and is regarded as a chronic slow growing disease (Gulsun et al., 2010; Thompson and McManus, 2002). Other studies showed higher prevalence of HD in certain age groups, including individuals aged 21 to 40 years with a prevalence rate of 47.8% and 46.7% (Zahawi et al., 1999; Salama et al., 2014), a rural–agricultural and semi-Bedouin population aged 11–20 with prevalence of about 40%, and a Bedouin population aged 31–40 years with a seroprevalence of 33% (Qaqish et al., 2003). In the Middle East, collective IgG seropositive rate was significantly different in the age groups of ≤19 (2.7%), 20–39 (8.3%), and ≥40 years (7.2%) (Galeh et al., 2018). The seroprevalence rate was similar in different areas of Al-Mafraq Governorate, and no significant correlation was noted among specific areas. The majority of study participants (45.5%) did not complete their secondary education, but there was no significant correlation between educational level and seroprevalence of HD. Many studies have indicated the importance of low educational level in facilitating transmission of infectious diseases, including HD (Abo-Shehada, 1993; Dowling et al., 2000; Seimenis, 2003). Despite the differences in the educational levels of study participants, almost none of them had any knowledge about HD, its causes, or its transmission methods. This study showed that 75.2% of participants reported no prior knowledge about HD, and 99.8% were not aware of the etiology of the disease. These results agree with previous observations regarding poor knowledge about the source of HD infection, the causative agent, and the transmission methods in a Jordanian population (Srinivas et al., 2016).

The only symptom that showed a significant association with positive IHA was weight loss. HD is mostly asymptomatic for many years and could lead to subsequent weight loss in the absence of other symptoms (Frider et al., 1999; Frider et al., 1988). Unexplained weight loss in individuals in endemic areas should raise the suspicion of HD among other causes. Among all possible risk factors included in this study, slaughtering sheep inside the house was the only risk factor that correlated significantly with positive IHA. The prevalence of HD in slaughtered animals from northern Jordan was higher in sheep compared to other animals, and the prevalence of HD in sheep increased with age to reach 100% in ewes of 10 years of age or older (Frider et al., 1999). Other possible risk factors including eating unwashed or uncooked vegetables, salads, and herbs; slaughtering sheep inside homes; and feeding dogs with the slaughtered remnants did not correlate with HD seroprevalence in this study. These findings are in agreement with other studies (Qaqish et al., 2003; Kern et al., 2004).

A valuable addition to this study would be testing all seropositive individuals with other more specific immunological test (Western blot), and performing imaging studies (ultrasound, chest X-ray, and CT-scan). Accordingly, a follow up was attempted to all seropositive individuals to perform imaging studies. However, most participants either did not agree to continue with the follow up step or communication was lost. The findings of this study highlight the importance of serological screening of high risk asymptomatic individuals using IHA. More specific tests and imaging studies would be required to confirm the diagnosis and to determine the clinical outcome of seropositive individuals.

## 5 Conclusions

Hydatid disease is an endemic disease in many areas of Jordan. Seroprevalence of HD in areas at elevated risk using IHA testing has not been investigated in Jordan. The seroprevalence of HD in Al-Mafraq city was 4.1%, which is higher than the rate reported by similar previous studies. Seropositivity correlates positively with unexplained weight loss and slaughtering sheep inside houses. Almost all participants were unaware about the etiology of the disease. IHA testing is a simple, cheap, and informative test for the screening of asymptomatic individuals in endemic areas. Unsanitary practices, including the slaughtering of animals inside houses, are still evident and would play an important role in HD transmission. Improving knowledge of the etiology and transmission of HD is important for its prevention. The implementation of appropriate control measures would decrease disease transmission and dissemination.

## Conflict of interest statement

All authors declare no conflicts of interest.

## Ethical approval

The study protocol was approved by the IRB at the Hashemite University (No: 1702184/440) and the Jordanian Ministry of Health (No: 17030).
